# Thiamine deficiency secondary to intestinal surgery leading to shoshin beriberi and Wernicke encephalopathy: a case report

**DOI:** 10.3389/fped.2025.1576805

**Published:** 2025-05-26

**Authors:** Peiyu Huang, Junyu Chen, Zhixuan Deng, Weiguo Yang

**Affiliations:** ^1^ Department of Pediatric Intensive Care Unit, Shenzhen Children’s Hospital, Shenzhen, China; ^2^ Hengyang Medical School, Institute of Cell Biology, University of South China, Hengyang, Hunan, China

**Keywords:** thiamine deficiency, shoshin beriberi, Wernicke encephalopathy, postoperative complications, neurological and cardiovascular involvement

## Abstract

**Purpose:**

Thiamine is an essential micronutrient, and its deficiency, also known as beriberi, can have significant clinical implications. Thiamine deficiency impairs oxidative metabolic pathways, leading to reduced adenosine triphosphate (ATP) synthesis and energy production. This deficiency primarily affects energy-demanding organs, such as the brain and heart, resulting in central and peripheral neuropathies, severe metabolic acidosis, or even circulatory failure. Due to its nonspecific clinical presentation, thiamine deficiency is often misdiagnosed or overlooked, making early recognition and intervention critical for improving outcomes.

**Case report:**

This report describes a case of shoshin beriberi with concurrent neurological involvement caused by thiamine deficiency following intestinal surgery. Remarkably, the patient's condition improved significantly with timely thiamine supplementation.

**Conclusion:**

In individuals at high risk of thiamine deficiency, such as those with prolonged fasting, gastrointestinal disorders, or chronic malnutrition, early identification and timely intervention are crucial for improving prognosis and reducing mortality. Early recognition and treatment are critical to reducing mortality associated with this condition.

## Introduction

Thiamine, also known as vitamin B1, is a vital micronutrient. Thiamine deficiency, commonly referred to as beriberi, can disrupt oxidative metabolic pathways, leading to reduced adenosine triphosphate (ATP) synthesis and diminished energy production (1–3). This deficit disproportionately affects organs with high energy demands, such as the brain and heart, resulting in central and peripheral neuropathy, severe metabolic acidosis, or circulatory failure (4–6).

Managing beriberi poses significant challenges for clinicians. Firstly, the clinical manifestations associated with thiamine deficiency are often nonspecific and may vary significantly when concurrent conditions are present (7, 8). Secondly, diagnostic methods to confirm thiamine deficiency are not routinely utilized, potentially delaying diagnosis and exacerbating adverse outcomes (9, 10). Lastly, the treatment strategies for this condition remain incompletely defined (7, 11).

This case report describes a pediatric patient who developed shoshin beriberi with neurological impairment following intestinal surgery and prolonged fasting without adequate supplementation of essential vitamins, including thiamine. By integrating the patient's clinical presentation and imaging findings, we explore the diagnostic challenges, pathophysiological mechanisms, and therapeutic strategies for thiamine deficiency. This study highlights the critical role of early recognition and timely intervention in improving prognosis and demonstrates the effectiveness of an individualized thiamine supplementation regimen in reversing life-threatening complications, providing valuable clinical insights for the screening and management of high-risk pediatric populations.

## Clinical data

The patient was a 14-year-old adolescent girl with a height of 170 cm and an admission weight of 39.8 kg, resulting in a BMI of 13.8, indicative of severe malnutrition. She was admitted to the hospital with a 20-day history of abdominal pain and vomiting, initially diagnosed with “intestinal obstruction.” The patient was diagnosed with intestinal developmental abnormalities in the neonatal period. At one month of age, due to duodenal and jejunal atresia with malrotation, the patient underwent intestinal resection, anastomosis, and adhesion release surgery. However, persistent duodenal obstruction required further surgical intervention at three months of age, where a side-to-side duodenojejunostomy was performed.

Prior to admission, the patient had undergone 20 days of fasting at an external hospital, receiving only glucose-containing IV fluids without parenteral nutrition or vitamin supplementation, with an unknown caloric intake. Upon transfer, she was admitted to the general surgery department and underwent laparoscopic Ladd's procedure and adhesion release. Postoperatively, she received fasting and gastrointestinal decompression therapy. On postoperative day(POD)2, the patient remained fasting while receiving total parenteral nutrition (TPN) at an initial energy intake of approximately 18.8 kcal/kg. However, due to an allergic reaction manifested as a rash after receiving parenteral nutrition, the nutritional regimen was gradually adjusted. Firstly, the intake of lipid emulsion was adjusted to 8.8 kcal/kg, and water-soluble vitamins were not supplemented. The patient had no known history of alcohol consumption or liver disease.

One week postoperatively, the patient developed malnutrition-associated liver dysfunction. By the second postoperative week, she exhibited hyponatremia, hypokalemia, metabolic acidosis, gastrointestinal and mucosal bleeding (notably involving the gastric mucosa, lips, tongue, and pharynx), pancytopenia, and elevated C-reactive protein (CRP) levels. Despite treatment with cefoperazone-sulbactam, electrolyte correction, and acid-base balance therapy, her condition showed limited improvement. On POD 22, the patient was transferred to the pediatric intensive care unit (PICU) for further management, the patient's weight had declined to 35.6 kg, with a BMI of 12.3, confirming a critical malnourished state.

### Postoperative course and clinical interventions

**Figure 1:**
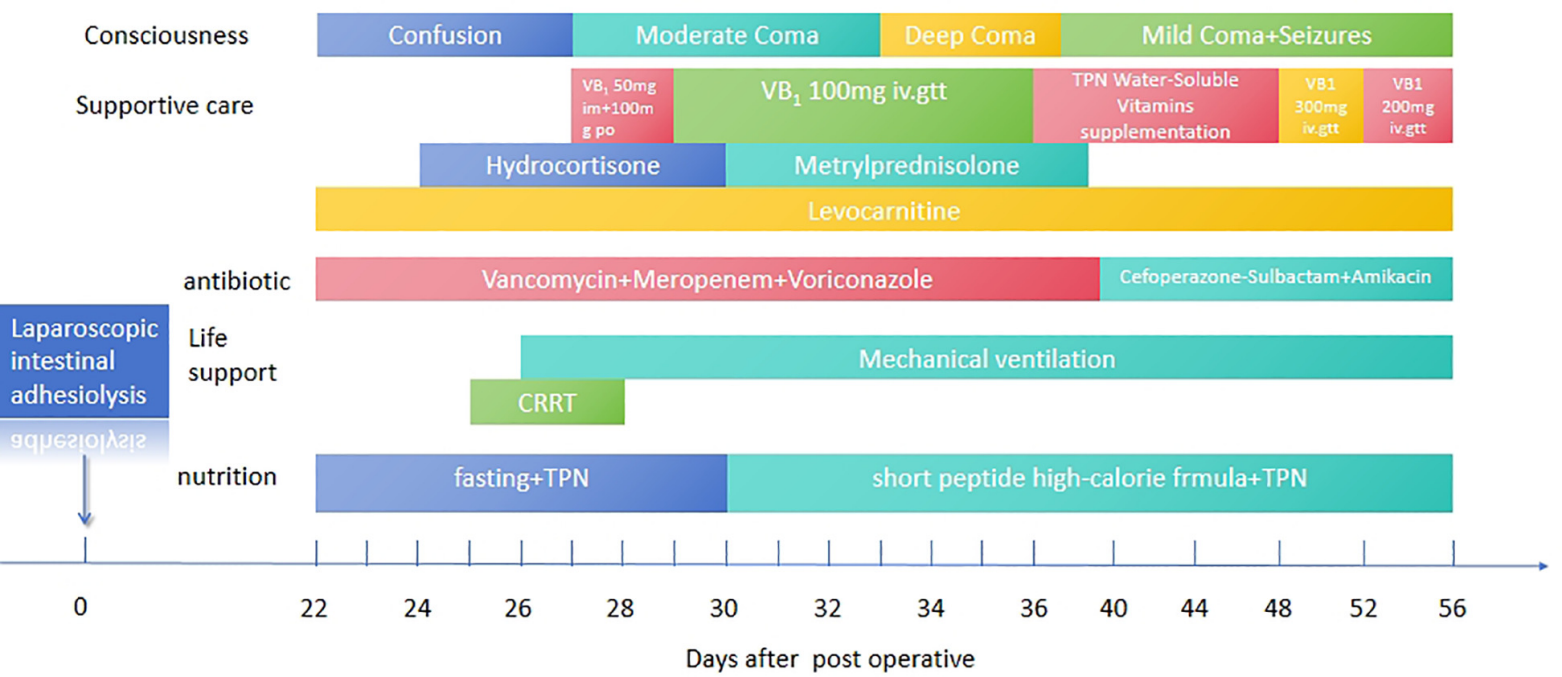
Clinical treatment timeline of the patient. The timeline summarizes key interventions and outcomes, including thiamine supplementation, extracorporeal therapies, and follow-up assessments, highlighting the progression and management of thiamine deficiency-related complications.

On POD 22, the pediatric patient was transferred to the Pediatric Intensive Care Unit (PICU) for intensive care management due to signs of shock. To optimize tissue perfusion, the patient received volume expansion therapy, acid-base correction, fluid resuscitation, and vasopressor support with low-dose dopamine and norepinephrine. Empirical broad-spectrum antimicrobial therapy was initiated with vancomycin, meropenem, and voriconazole, alongside metabolic support with levocarnitine and creatine phosphate sodium. The patient was supplemented with vitamins B6, B12, C, and K1, as well as potassium, calcium, and phosphorus. Hydrocortisone was administered for its anti-inflammatory effects. Neurological assessment via lumbar puncture and electroencephalography (EEG) yielded no significant abnormalities (Figure 1).

By POD 25, the patient exhibited progressive, unexplained lactic acidosis, with serum lactate levels peaking at 20 mmol/l. Continuous renal replacement therapy (CRRT) was initiated to facilitate lactate clearance. On POD 26, worsening hypercapnia led to a deterioration in the patient's level of consciousness, progressing from confusion to coma, accompanied by hemodynamic instability. Endotracheal intubation and mechanical ventilation were implemented, with additional administration of epinephrine for blood pressure support. Despite these measures, the patient's blood pressure declined further, reaching a nadir of 65/40 mmHg. The combination of dopamine and dobutamine was ineffective in restoring hemodynamic stability. However, empirical supplementation with water-soluble vitamins resulted in a notable improvement in blood pressure and a subsequent decline in serum lactate levels.

**Figure 2:**
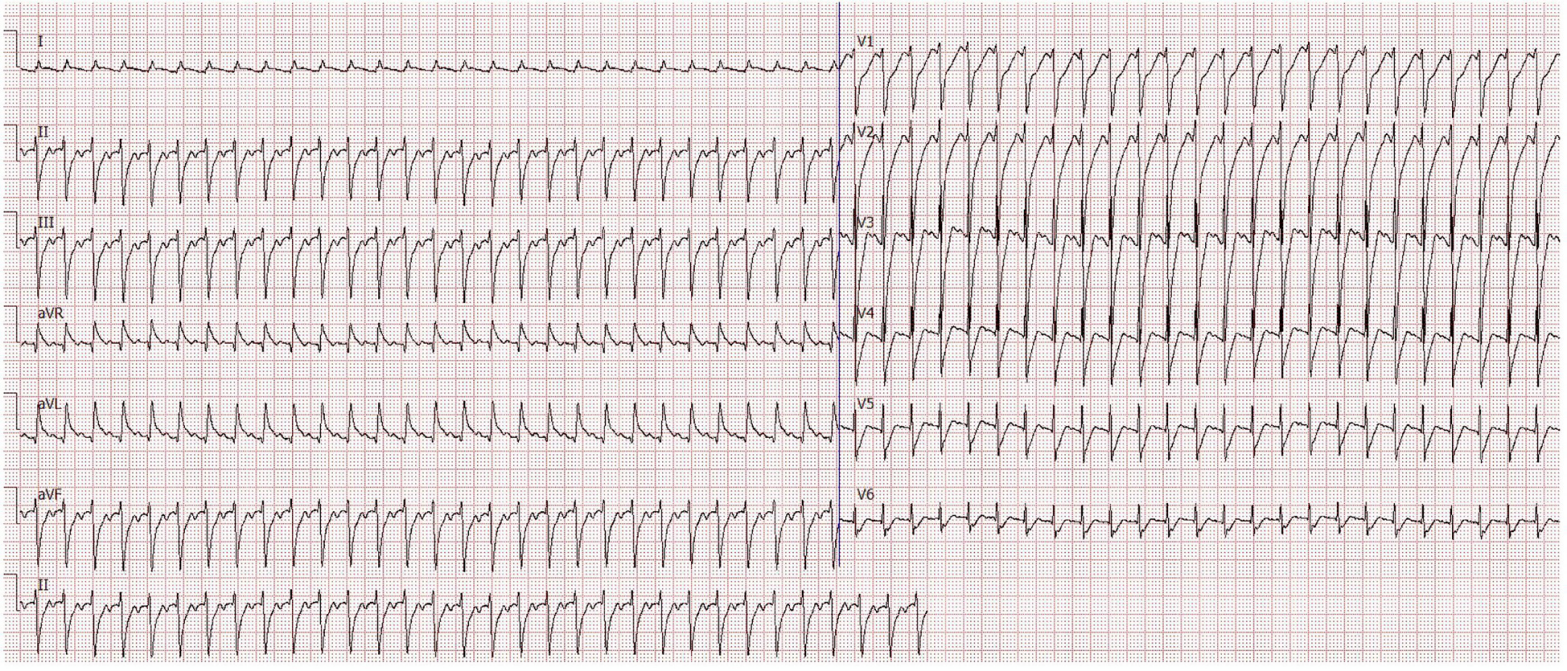
Electrocardiogram during paroxysmal supraventricular tachycardia (PSVT). The ECG shows characteristic features of PSVT observed during acute cardiovascular decompensation, highlighting severe cardiovascular dysfunction in the patient.

Despite hemodynamic stabilization, the patient exhibited persistent tachycardia (150–160 beats per minute) and maintained low blood pressure. One day later, a sudden increase in heart rate to 190 beats per minute was noted, along with polyuria. Bedside electrocardiography (ECG) indicated paroxysmal supraventricular tachycardia (PSVT) with ST-T wave abnormalities, while echocardiography revealed myocardial echogenic changes suggestive of myocardial injury (Figure 2). Left ventricular systolic function was at the lower limit of normal, whereas right atrial pressure indicators were elevated (inferior vena cava diameter: 20 mm, collapse index: ∼11%). Fluid resuscitation and tapering of catecholamines failed to alleviate the tachycardia. Laboratory findings revealed severe hypokalemia (K^+^; = 2.54 mmol/l, reference range: 3.7–5.2 mmol/l) and hypophosphatemia (P^3^^+^; < 0.21 mmol/l, reference range: 1.03–1.86 mmol/l), suggesting the development of refeeding syndrome following vitamin supplementation. The patient subsequently experienced malignant arrhythmias, necessitating two episodes of electrical cardioversion/defibrillation.

**Figure 3:**
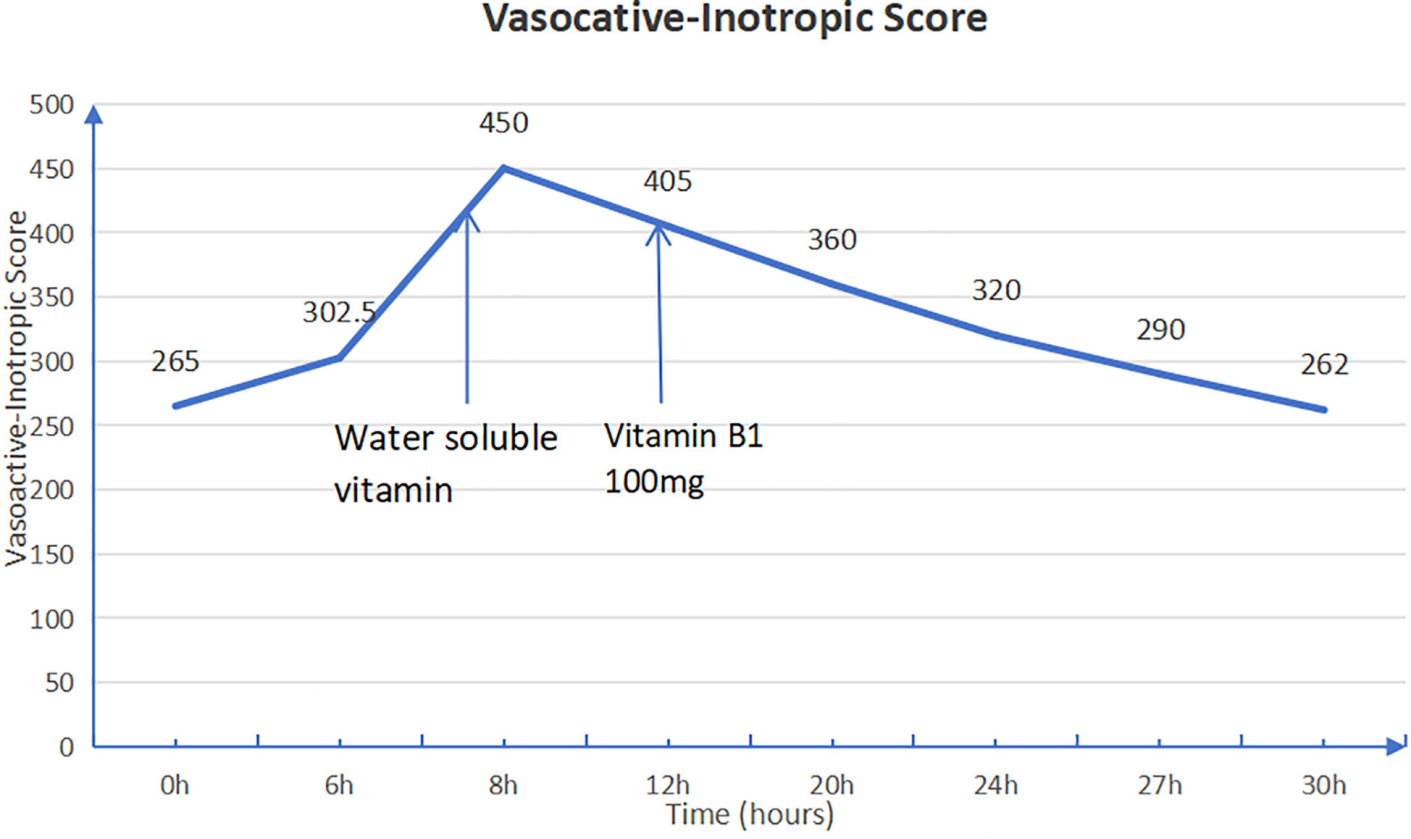
Vasoactive-inotropic score (VIS) before and after thiamine administration. The VIS is calculated as dopamine dose [μg/kg/min] + dobutamine dose [μg/kg/min] + 100 × epinephrine dose [μg/kg/min] + 10 × milrinone dose [μg/kg/min] + 10,000 × vasopressin dose [U/kg/min] + 100 × norepinephrine dose [μg/kg/min]. A significant reduction in VIS after thiamine therapy indicates improved cardiovascular function.

Given the history of multiple intestinal surgeries, prolonged fasting, malabsorption, and insufficient vitamin supplementation in total parenteral nutrition (TPN), the patient exhibited persistent lactic acidosis and neurological impairment. Empirical high-dose intravenous thiamine therapy (100 mg orally and 50 mg intramuscularly) was initiated. Following thiamine supplementation, the patient's cardiac rhythm reverted to sinus rhythm, allowing for the gradual weaning of vasoactive medications. Intravenous thiamine (100 mg daily) was continued for one week, after which all vasopressor support was successfully discontinued (Figure 3).

### Neurological and radiological findings

**Figure 4:**
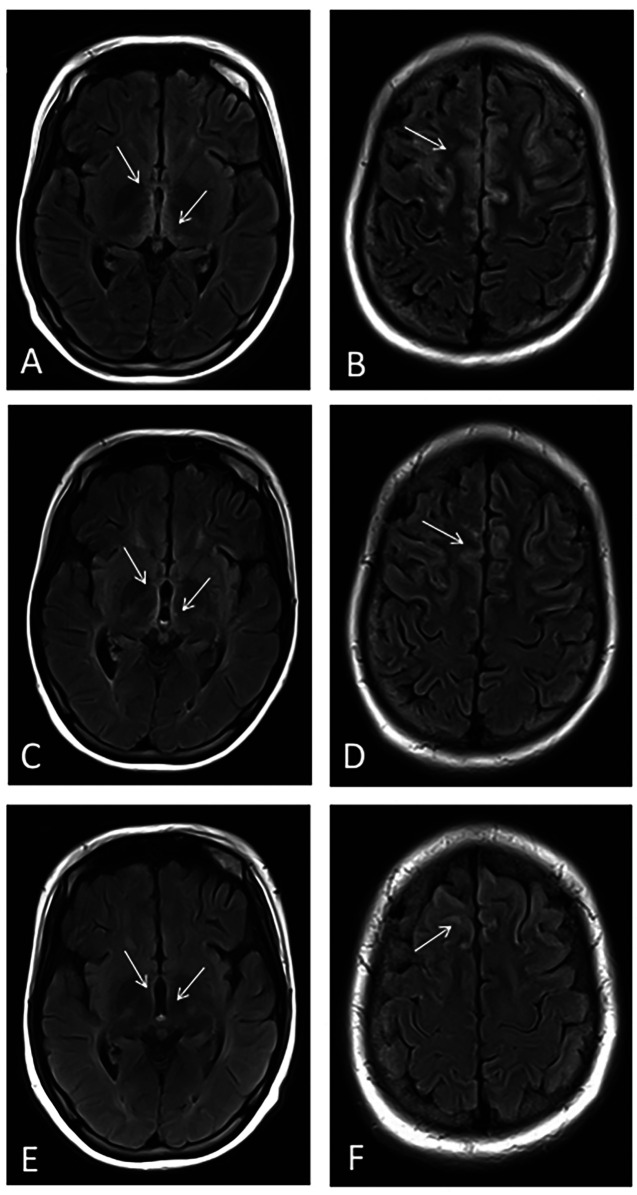
Dynamic changes in brain MRI findings. **(A,B)** MRI at presentation showing symmetrical abnormal signals in the bilateral frontal cortical regions, periventricular thalamus, mammillary bodies, and dorsal medulla. **(C,D)** MRI one month after treatment demonstrating reduced abnormalities. **(E,F)** MRI three months after treatment showing further improvement, including the resolution of periaqueductal hyperintensities.

After achieving hemodynamic stability, further neuroimaging via brain magnetic resonance imaging (MRI) revealed symmetrical signal abnormalities in the bilateral frontal cortex, periventricular dorsal thalamus, mammillary bodies, and dorsal medulla, characterized by cytotoxic edema. Based on these characteristic imaging findings and the patient's clinical history, a diagnosis of Wernicke encephalopathy (WE) was strongly suspected (Figures 4A,B).

Therapeutic management continued with intravenous vitamin B1 (100 mg daily) until POD 36. Due to serum vitamin B1 levels exceeding the reference range, intravenous supplementation was discontinued and replaced with a lower-dose intravenous and oral regimen.

On POD 40, the patient developed recurrent seizures, manifesting as head nodding, bilateral upper limb tremors, and eyelid myoclonus, accompanied by hypertension and pupil dilation. Antiepileptic treatment was initiated with midazolam infusion and intravenous levetiracetam, alongside continued micronutrient and vitamin supplementation. However, seizure activity remained frequent. High-dose intravenous thiamine (300 mg TID) was administered as a loading regimen for two consecutive days, followed by 200 mg QD for maintenance over the next 3–5 days. This resulted in a marked reduction in seizure frequency. Dynamic monitoring of serum vitamin B1 levels (ranging from 73.83 to 4.06 nmol/l) guided dose adjustments.

One month later, follow-up brain MRI demonstrated partial resolution of the bilateral frontal cortical, periventricular dorsal thalamic, mammillary body, and dorsal medulla abnormalities observed previously (Figures 4C–F).

### Long-term follow-up and prognosis

The patient received multiple sessions of hyperbaric oxygen therapy as part of neurorehabilitation. At six months post-treatment, the patient remained in a comatose state but exhibited responses to external stimuli, such as tear production and facial expressions, with occasional involuntary finger movements. By nine months post-treatment, the patient demonstrated improved awareness of the external environment, showing emotional responses (crying or smiling) to basic communication. Additionally, the patient was able to follow simple commands, such as mouth opening and coughing, and exhibited a strong desire for oral intake.

## Discussion

Thiamine, or vitamin B1, deficiency, commonly known as beriberi, is traditionally categorized into two main types. The first is “dry beriberi,” characterized by central and peripheral neuropathy, such as Wernicke encephalopathy (WE) (12). The second is “wet beriberi,” primarily presenting with cardiovascular symptoms and signs (13). The case presented here primarily demonstrated an acute variant termed Shoshin beriberi, which is marked by severe circulatory collapse, metabolic acidosis, and cardiovascular failure. Additionally, the patient exhibited features associated with traditional types of beriberi, such as WE (14).

Previous studies have reported that thiamine deficiency occurs in 20% of critically ill patients and in 20%–70% of patients with sepsis (1, 15). Thiamine deficiency disrupts oxidative metabolic pathways, reducing ATP synthesis and energy production, thereby impacting high-energy-demand organs such as the brain and heart (1–3). In the brain, thiamine deficiency impairs energy supply, increases oxidative stress, and alters neurotransmitter transmission (16). In the heart, impaired aerobic metabolism can lead to arrhythmias, cardiac dysfunction, and progressive lactic acidosis (17, 18). Thiamine-dependent nitric oxide synthase deficiency exacerbates endothelial dysfunction, potentially triggering pulmonary hypertension (19, 20).

Clinical presentations of thiamine deficiency disorders (TDDs) are often nonspecific and highly variable (7). In pediatric patients with thiamine deficiency (TD) in high-income countries, 42% of acute cases present with either typical or atypical Wernicke encephalopathy (WE). Other common acute manifestations include hyperlactatemia (37%), altered consciousness (28%), and heart failure (21, 22). Diagnostic methods include serum thiamine level measurement, urinary thiamine level assessment, erythrocyte transketolase activity, and thiamine pyrophosphate analysis (7). However, these tests are limited by availability and timeliness.

Neurological symptoms in WE are typically associated with characteristic acute-phase MRI findings, such as symmetrical T2WI hyperintensities around the third ventricle and aqueduct, as well as in the thalami and brainstem (9). In this case, early-phase MRI findings corresponded to these characteristic changes, supporting the diagnosis of beriberi. Notably, cortical involvement was also observed in this pediatric case, indicating a relatively poor prognosis, often accompanied by complex symptoms such as impaired consciousness and seizures, which aligns with the clinical manifestations of this patient (23).

Thiamine deficiency-associated lactic acidosis is classified as type B lactic acidosis. In pediatric cases, common causes include inadequate enteral or parenteral nutrition (24–27). Type B lactic acidosis is often challenging to recognize (28). When patients present with shock, elevated lactate levels, and signs of infection, clinicians typically prioritize a diagnosis of septic shock. However, in high-risk populations, such as those with prolonged parenteral nutrition, inadequate enteral nutrition, or a history of malignancy, persistent elevated blood lactate levels despite circulatory improvement following treatment should raise suspicion for Type B lactic acidosis (29, 30). Given the delayed turnaround time of relevant laboratory tests, a high index of clinical suspicion is essential, and urgent empirical treatment should be initiated preemptively (30).

In patients with cardiovascular-type thiamine deficiency, the response to thiamine administration is rapid (31, 32). Clinical signs such as shock, irritability, and tachycardia typically improve within a few hours, while respiratory rate and liver size demonstrate significant reductions within 48 h following supplementation (30, 32, 33). In exclusively breastfed infants diagnosed with cardiac beriberi, intramuscular administration of thiamine at a dose of 75 mg twice daily for five days led to the reversal of echocardiographic abnormalities within 2–3 weeks (34). However, whether variations in thiamine dosage and treatment duration significantly impact survival outcomes remains unclear, as no dedicated studies have addressed this issue (35).

In contrast to cardiovascular thiamine deficiency disorders (TDDs), neurological involvement often necessitates higher thiamine doses and a prolonged recovery period (36). A retrospective cranial ultrasound study investigating thiamine-responsive encephalopathy in hospitalized infants identified 58 cases, of which 41 exhibited abnormal ultrasound findings (37). Among these infants, 63% (*n* = 26) demonstrated gradual regression of basal ganglia lesions, requiring 4–8 weeks for full resolution. However, 37% exhibited persistent basal ganglia hyperechogenicity, and 15% developed basal ganglia atrophy with ventricular enlargement on follow-up imaging. All infants received an initial intravenous thiamine dose of 100 mg upon admission, followed by oral thiamine supplementation for six weeks post-discharge. The need for high-dose thiamine therapy in severe neurological involvement is further exemplified by a case of Wernicke encephalopathy (WE) secondary to prolonged malnutrition due to neuroblastoma treatment. In this case, thiamine supplementation at 100 mg/day failed to produce clinical improvement, whereas high-dose thiamine therapy (500 mg TID, total 1,500 mg/day) led to symptom resolution, with MRI normalization observed after two months of treatment (11).

According to the European Federation of Neurological Societies (EFNS) guidelines, the recommended treatment for WE is intravenous thiamine at a dose of 200 mg TID, continued until no further clinical improvement is observed (38). Additionally, thiamine must be administered before glucose infusion, as glucose administration can precipitate Wernicke encephalopathy by exacerbating thiamine depletion (38, 39).

In the present case, the patient's prolonged impairment of consciousness may be attributed to insufficient thiamine dosing and delayed administration for neurological involvement. Given the preoperative presence of severe malnutrition, earlier or prophylactic thiamine supplementation may have improved the overall prognosis. These findings underscore the importance of multidisciplinary perioperative management for high-risk pediatric patients, ensuring timely vitamin B1 supplementation to prevent neurological complications and optimize clinical outcomes.

In critically ill patients, the potential effects of extracorporeal support therapies, including continuous renal replacement therapy (CRRT) and extracorporeal membrane oxygenation (ECMO), on thiamine metabolism should be considered (40). A study investigating critically ill pediatric patients demonstrated a significant reduction in serum folate concentrations following CRRT (41). Although this study did not directly assess thiamine levels, folate, as another water-soluble B vitamin, suggests a potential parallel mechanism in which CRRT may enhance thiamine clearance.

According to the European Society for Clinical Nutrition and Metabolism (ESPEN) guidelines, CRRT is associated with increased losses of water-soluble micronutrients, including B vitamins (42–44). In our case, the patient underwent CRRT and developed hypotension within two days of treatment, which may indicate that CRRT further exacerbated thiamine depletion, contributing to cardiovascular collapse. Therefore, during CRRT, it is recommended to increase intravenous thiamine supplementation by 100% to 200% to compensate for potential losses and mitigate the risk of thiamine deficiency-related complications (45).

## Conclusions

In patients at high risk of thiamine deficiency, including those with prolonged fasting, gastrointestinal disorders, or chronic malnutrition, a high index of clinical suspicion is essential. Prompt empirical treatment should be initiated, as early intervention may reverse life-threatening complications. The dosage and duration of thiamine therapy should be tailored based on the patient's clinical presentation and the extent of organ involvement to optimize therapeutic outcomes.

## Data Availability

The original contributions presented in the study are included in the article/Supplementary Material, further inquiries can be directed to the corresponding author.
